# Development of medical freezing measures in women during the last decade from 2014 to 2023: registry data of the tri-national network *Ferti*PROTEKT

**DOI:** 10.1007/s00404-025-08253-7

**Published:** 2026-01-13

**Authors:** Angela Vidal, Verena Nordhoff, Moritz Suerdieck, Janna Pape, Michael von Wolff

**Affiliations:** 1 Division of Gynecological Endocrinology and Reproductive Medicine, University Women’s Hospital, Theodor-Kocher-Haus, Friedbühlstrasse 19, CH-3010 Bern, Switzerland; 2 Centre of Reproductive Medicine and Andrology, University of Münster, Münster, Germany; 3 Gyn-A.R.T. AG, Zurich, Switzerland

**Keywords:** Fertility preservation, Ovarian tissue, Ovarian stimulation, Oocytes, *Ferti*PROTEKT

## Abstract

**Research question:**

To what extent have fertility preservation interventions evolved between 2014 and 2023, and what factors have influenced changes in their utilization and prevalence?

**Design:**

Based on the *Ferti*PROTEKT registry, comprising 163 centres across Germany, Austria, and parts of Switzerland, the quantitative development of ovarian stimulation for oocyte cryopreservation and ovarian tissue cryopreservation was evaluated from 2014 to 2023. Analyses were stratified according to the kind of participating centre, patient age, and the spectrum of underlying diseases. In addition, data were statistically compared for the periods 2014/2015 (P1) and 2022/2023 (P2).

**Results:**

Approximately 14,000 women received counselling across all three countries between 2014 and 2023. Among these, 3,996 females underwent ovarian stimulation for oocyte cryopreservation, and 3,478 underwent ovarian tissue cryopreservation. The number of oocyte cryopreservation cycles increased substantially from P1 to P2, whereas the number of ovarian tissue cryopreservation procedures remained relatively stable. The increase in oocyte cryopreservation was substantially greater in private centres (197% increase: 308 to 916 cycles) compared to public institutions (39% increase: 818 to 1,136 cycles; p < 0.001). The rise in oocyte cryopreservation cycles parallels an increase in breast cancer cases presenting for fertility preservation; this temporal coincidence suggests a potential association but does not establish causation. The predominance of breast cancer patients also influenced the age distribution of oocyte cryopreservation cases. Among oocyte cryopreservation procedures, absolute numbers increased across all age groups up to 40 years, with the largest absolute increase in women aged 31–40 years (212 to 732 cycles, 245% relative increase).The overall age distribution of procedures changed only slightly, although younger patients were more likely to undergo ovarian tissue cryopreservation. Additionally, new indications such as endometriosis and gender dysphoria have become increasingly relevant over the past 5 years.

**Conclusion:**

The number and distribution of fertility preservation procedures have changed notably during the last decade, driven primarily by shifts in the reimbursement strategies and the type of centres providing care. These developments should be carefully considered in the future design and implementation of fertility preservation programmes. However, decisions regarding specific fertility-preserving interventions must also be guided by scientific evidence.

**Supplementary Information:**

The online version contains supplementary material available at 10.1007/s00404-025-08253-7.

## What does this study add to the clinical work

**Table:** 

This study informs clinical practice by demonstrating that the evolution and uptake of fertility preservation interventions are primarily determined by reimbursement policies and categories of healthcare institutions.
It emphasizes the need to integrate these systemic factors into evidence-based patient counselling and program design, while providing projections to guide the strategic planning and optimization of fertility preservation services.

## Introduction

Fertility preservation has gained increasing importance due to improved long-term survival rates among cancer patients [1–3]. Preserving fertility in women presents particular medical challenges, as it may require delaying cancer treatment and can involve health risks for the patient. Available fertility preservation methods include laparoscopic removal and cryopreservation of ovarian tissue, ovarian stimulation and oocyte cryopreservation, as well as the administration of gonadotropic releasing hormone (GnRH) analogues [3]. The choice of method depends on factors such as the patient’s age, the gonadotoxic potential of the planned treatments, and the time available before initiation of therapy [4–10].

The development of cryopreservation techniques and indications over the past two decades has been remarkable. Initially focused on oncological patients, fertility preservation is now increasingly relevant for non-oncological indications [11–14].

Fertility preservation requires a multidisciplinary approach before and after gonadotoxic treatment to ensure integration into complex, multimodal cancer care. It also presents a significant challenge to national health systems. Fertility preservation services must be universally and promptly accessible and, ideally, covered by public health systems. These requirements can only be fulfilled through coordinated treatment protocols and large collaborative networks of fertility preservation centres embedded within oncological care structures [15].

The *Fert*iPROTEKT network (www.FertiPROTEKT.com) has played a pioneering role in developing collaborative treatment models that bring together oncologists, reproductive specialists, and other healthcare providers to ensure timely fertility counselling and access to fertility preservation options prior to treatment [13]. Established in 2006, the network initially operated in Germany and expanded to Austria and Switzerland. *Fert*iPROTEKT has previously published longitudinal analyses of approximately 5,000 patients covering the period from 2007 to 2013 [13], providing valuable insights into the range and effectiveness of fertility preservation strategies [16, 17].

Data from representative multinational registries are essential for understanding the current landscape of fertility preservation, improving clinical practices, and guiding the adaptation of strategies in response to evolving factors. These include the refinement of cryopreservation techniques that have enhanced the viability and functionality of preserved reproductive material, political discussions regarding treatment funding, and external events such as the COVID-19 pandemic, which has impacted access to fertility-related care [18–21].

Building on the first longitudinal analysis of *Fert*iPROTEKT covering the years 2007 to 2013, the present study aimed to conduct an in-depth follow-up evaluation of the development of fertility preservation strategies over the subsequent decade, from 2014 to 2023.

The objective of this study was to analyze the evolution of medical cryopreservation practices, specifically ovarian stimulation and ovarian tissue cryopreservation. Particular attention was given to trends in indications, procedures, and outcomes, with the aim of understanding how clinical guidelines and decision-making have adapted to the latest scientific evidence, and to support healthcare professionals in the establishment and optimization of current or future fertility preservation programs.

## Materials and methods

### The *Fert*iPROTEKT network

**Fig. 1:**
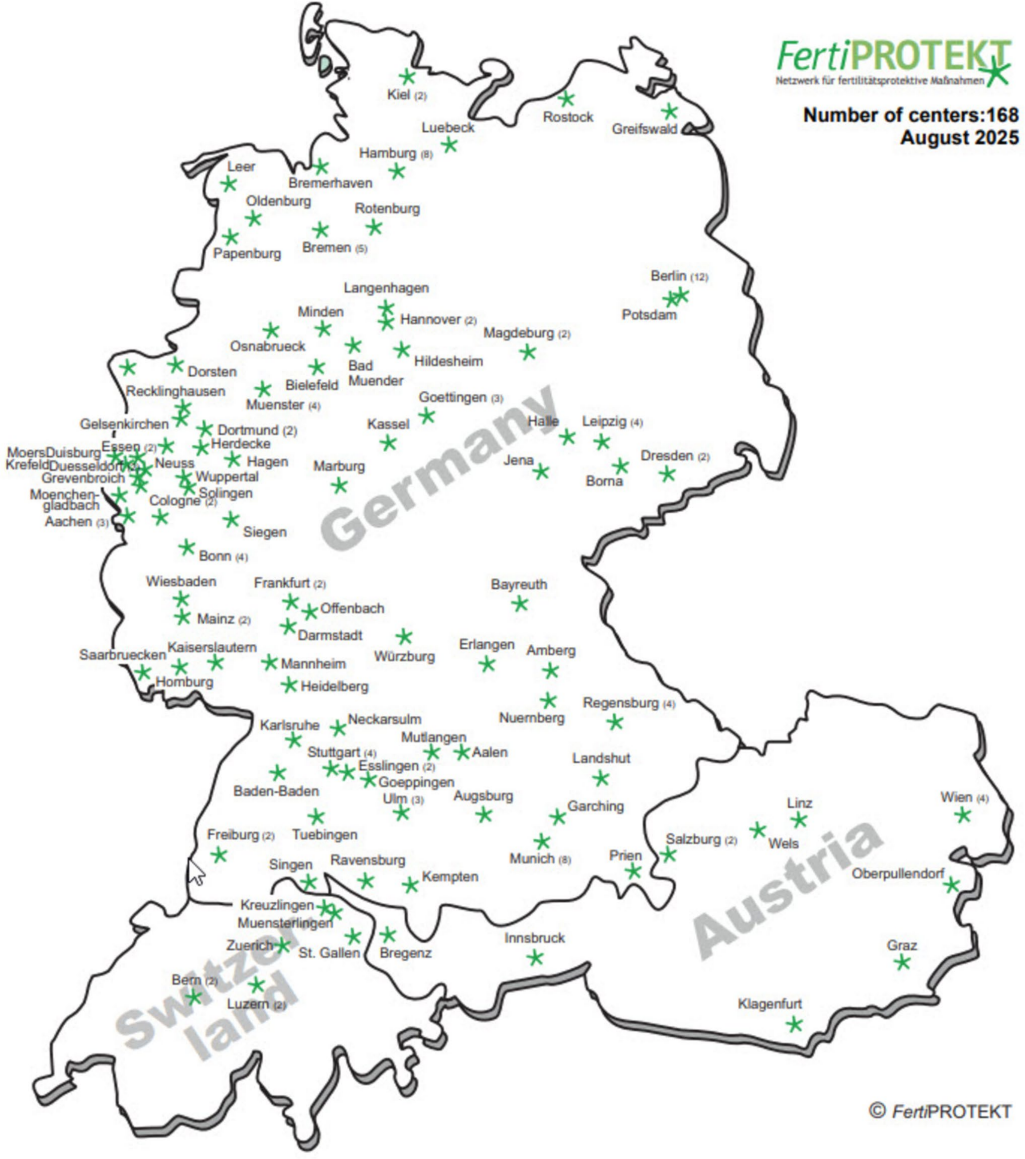
*Fert*iPROTEKT network centers (N = 167) across Germany, Austria, and Switzerland (©FertiPROTEKT)

The tri-national *Fert*iPROTEKT network was established in 2006 with the aim of advancing fertility preservation techniques, initially in Germany and subsequently expanding to the neighbouring German-speaking countries such as Austria and Switzerland (Fig. 1).

The network’s primary objective is to facilitate the scientific evaluation and development of fertility preservation technologies across university hospitals, private clinics, and outpatient facilities. Drawing on its collective experience, expertise, and recommendations at both national and international levels [13–16, 22–24], the network contributes to the advancement of fertility preservation measures and the formulation of clinical recommendations and guidelines [16, 17, 23]. The network’s website (www.FertiPROTEKT.com) serves as a comprehensive resource for both professionals and patients involved in fertility preservation. It provides comprehensive information on current clinical practices and facilitates access to clinics offering counselling and treatment options across Germany, Austria, and Switzerland, while acknowledging the influence of evolving health policy frameworks and economic factors on the accessibility and sustainability of fertility preservation services.

The *Fert*iPROTEKT registry records patient-level data, including oncological diagnosis and, where applicable, the fertility preservation techniques employed. Individual centres enter these data into secure online database software ASKIMED. An annual evaluation is conducted, and the results are presented at bi-annual workshops organized by the network. Summary data are made publicly accessible through the *Fert*iPROTEKT website and German IVF Register (Deutsches IVF-Register [D.I.R]) [25].

### Study population

We conducted a retrospective cohort study using data from the *Fert*iPROTEKT registry. Between 2014 and 2023, approximately 14,000 women received fertility counselling across 162 centers in Germany, Austria, and Switzerland.

A two-time 24-month period comparison was performed, with the first period spanning from 2014 to 2015 (P1) and the second covering 2022–2023 (P2). The study population was subsequently stratified by age groups, disease type (oncological vs. non-oncological), and centre type (private vs. public). Cases with incomplete or missing medical records were excluded from the analysis.

### Outcomes

The primary outcome was to evaluate changes in the incidence and utilization of fertility preservation techniques among the *Fert*iPROTEKT network, specifically ovarian stimulation followed by oocyte cryopreservation and ovarian tissue cryopreservation. This was assessed through a comparative analysis of the two defined periods: 2014 (P1) and 2022–2023 (P2).

Secondary outcomes included the distribution of procedures by medical condition (oncological vs. non-oncological), age-specific trends in the application of fertility preservation techniques, and differences in utilization patterns between university-affiliated and non-university centres.

### Statistics

Descriptive statistics were employed to characterize temporal trends in fertility preservation practices. Data are presented as absolute numbers and percentages. Categorical variables were compared between the two time periods (2014/2015 vs. 2022/2023) using chi-square tests. A two-sided p-value < 0.05 was considered statistically significant. All analyses were performed using (R version 2025.09.2 + 418). Missing centre-type data (n = 21 [4.4%] for oocyte cryopreservation in 2014/2015 and n = 21 [3.0%] for ovarian tissue cryopreservation in 2014/2015) were excluded from the respective centre-type stratified analyses but included in overall trend analyses. Complete case analysis was used; no data imputation was performed.

## Results

### Patient characteristics

Between 2014 and 2023, approximately 14,000 women received fertility counselling across the three participating countries. Of these, 3,996 women underwent ovarian stimulation for oocyte cryopreservation, and ovarian tissue cryopreservation was performed in 3,478 women.

### Ovarian stimulation and ovarian tissue freezing interventions

**Fig. 2:**
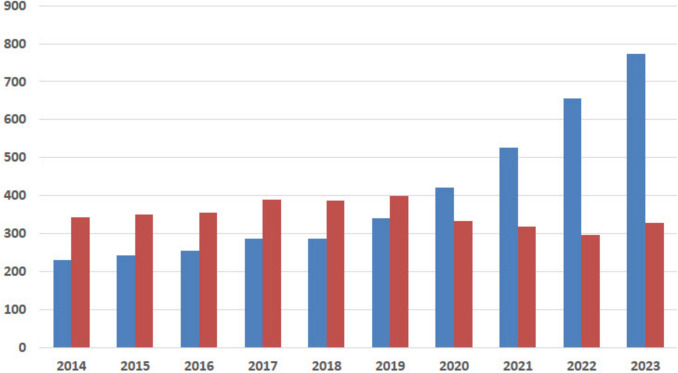
Trends in ovarian stimulation and ovarian tissue cryopreservation interventions from 2014 to 2023, data from the *Fert*iPROTEKT network (ovarian tissue cryopreservation interventions are represented by red bars, and ovarian stimulation procedures by blue bars; the x-axis depicts the respective years (2014–2023), the y-axis shows the total number of interventions)

**Fig. 3:**
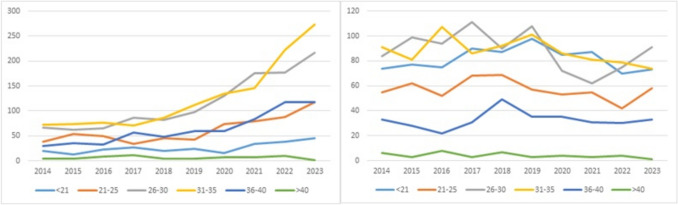
Age-based distribution of ovarian stimulation and ovarian tissue cryopreservation interventions, data from the *Fert*iPROTEKT network (the x-axis depicts the respective years (2014–2023), the y-axis shows the total number of interventions)

A significant increase in the number of ovarian stimulations was observed over the study period, whereas the number of ovarian tissue cryopreservation procedures showed a slight decline (Fig. 2). With regard to age distribution, the number of ovarian stimulation procedures increased across all age groups up to the age of 40, with the most pronounced rise seen among women aged 31–35 years. In contrast, ovarian tissue procedures declined with increasing age, with the exception of women under 21 years, in whom a moderate increase was observed (Fig. 3).

**Fig. 4:**
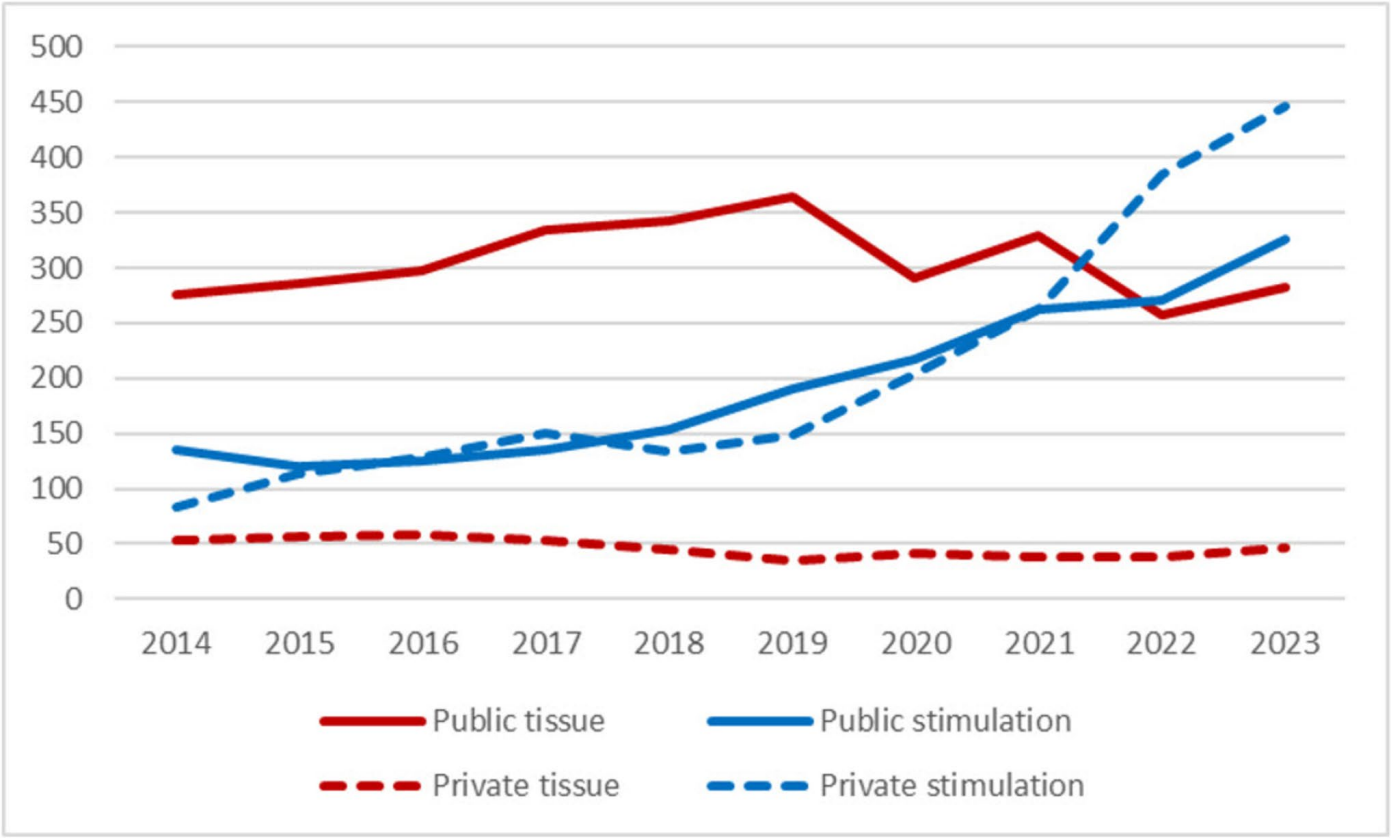
Ovarian stimulation and ovarian tissue cryopreservation interventions in public and private centres, data from the *Fert*iPROTEKT network (ovarian tissue cryopreservation is represented by red lines, and ovarian stimulation by blue lines; solid lines indicate public centres, while dashed lines represent private centres; the x-axis depicts the respective years (2014–2023), the y-axis shows the total number of interventions)

A significant rise in the number of ovarian stimulation cycles was recorded in private fertility centres, whereas public hospitals continued to perform the majority of ovarian tissue cryopreservation procedures, thereby maintaining service provision in this domain (Fig. 4).

### Fertility-preservation treatments in relation to patient characteristics

**Fig. 5:**
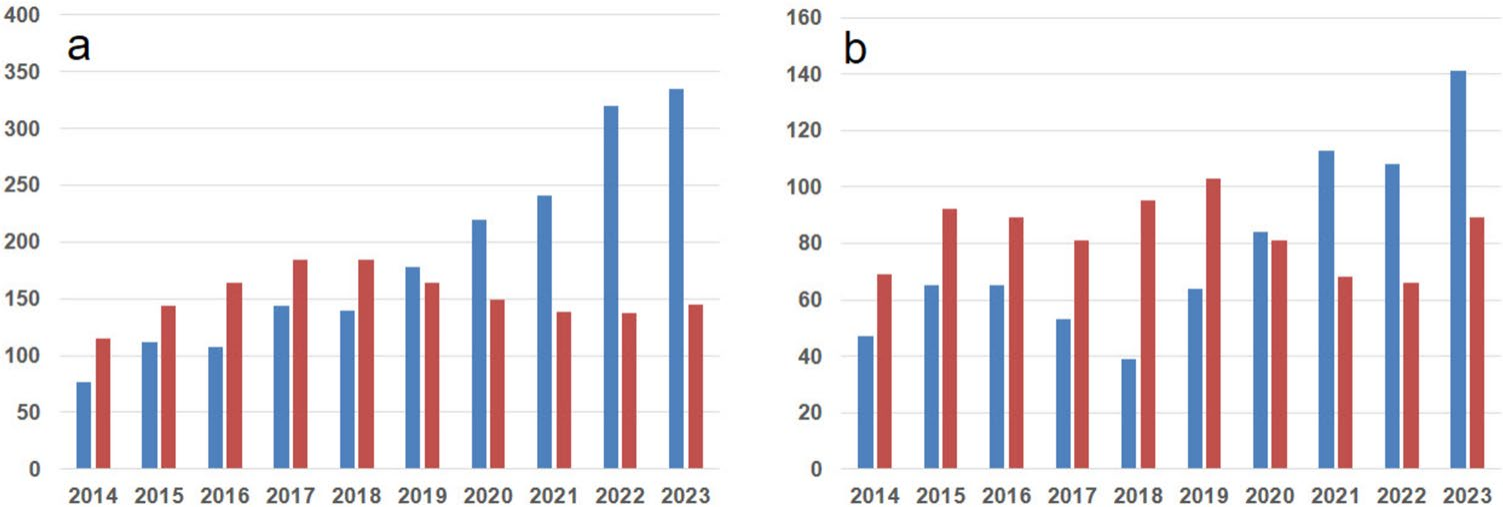
Ovarian stimulation and ovarian tissue cryopreservation interventions in patients with breast cancer (**a**) and lymphoma (**b**), data from the *Fert*iPROTEKT network (ovarian tissue cryopreservation is represented by red bars and ovarian stimulation by blue bars; the x-axis depicts the respective years (2014–2023), the y-axis shows the total number of interventions)

**Fig. 6:**
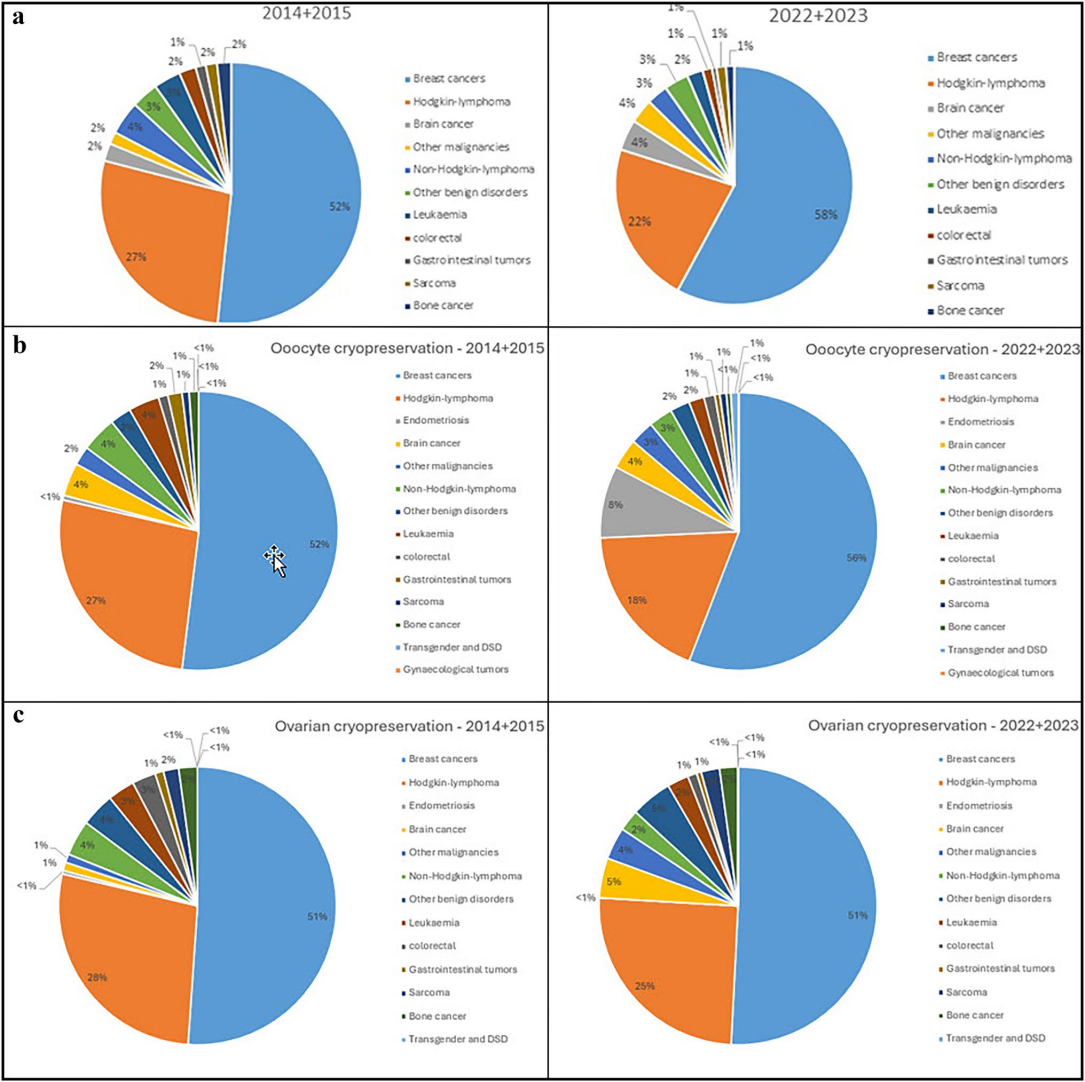
Spectrum of underlying diseases in all patients (**a**) in patients undergoing ovarian stimulation (**b**), and in patients undergoing ovarian tissue freezing (**c**) in 2014/2015 (P1) and 2022/2023 (P2). Data derived from the FertiPROTEKT network

The distribution of malignant diseases leading to fertility preservation remained broadly consistent over the study period. However, specific trends were noted within certain disease groups. Among women with breast cancer—the largest patient group (Fig. 6)—use of ovarian stimulation and subsequent oocyte cryopreservation increased significantly over time (Fig. 5). This trend likely represents the main driver of the overall increase in fertility preservation procedures shown in Fig. 2. A similar, though less pronounced, increase in ovarian stimulation procedures was observed among lymphoma patients, the second most common indication (Fig. 6). Nevertheless, ovarian tissue cryopreservation remained an important option for this group (Tables 1, 2).

**Table 1:** Overview of the reimbursement of fertility-preserving measures for women in Germany, Austria, and Switzerland

Criteria	Germany	Austria	Switzerland
Reimbursement of fertility preservation before cancer therapy	Yes	No	Yes
Lower age limit	From puberty	IVF Fund: from 18 years	Post-pubertal
Upper age limit	Up to 40 years	IVF Fund: up to 40 years	Up to 40 years
Criteria	Benign and malignant diseases that may lead to fertility loss Exception: urner syndrome Ovarian stimulation in hormone receptor–positive tumors (contraindication/restricted approval) Adolescents under 18 (restricted approval)	Partnership, indication, health insurance, main residence of one partner, age limits, citizenship (or residence permit)	Malignant and benign diseases requiring chemo- or radiotherapy, with a risk of amenorrhea > 20%
Techniques	Ovarian stimulation and cryopreservation of oocytes Removal and cryopreservation of ovarian tissue Storage up to the 40th year of age	–	Ovarian stimulation and cryopreservation of oocytes Removal and cryopreservation of ovarian tissue Transplantation of ovarian tissue Storage up to 10 years
Reimbursement of GnRH analogues	No	Upon request and subject to chief medical approval by the health insurance	No
Sufficient cost coverage of measures	No	No	Yes
Reimbursement of fertility preservation after cancer therapy	No (possibly case-by-case decision)	No	No

**Table 2:** Overview of the reimbursement of fertility-preserving measures for men in Germany, Austria, and Switzerland

Criteria	Germany	Austria	Switzerland
Reimbursement of fertility preservation before cancer therapy	Yes	No	Yes
Lower age limit	From puberty	IVF Fund: from 18 years	Post-pubertal
Upper age limit	Up to 50 years	IVF Fund: up to 50 years	Up to 40 years
Criteria	Benign and malignant diseases that may lead to fertility impairment	Partnership, indication, health insurance, main residence of one partner, age limits, citizenship (or residence permit)	Malignant and benign diseases requiring chemo- or radiotherapy, with a risk of azoospermia > 20%
Techniques	Cryopreservation of sperm Biopsies and cryopreservation of testicular tissue Storage up to the 50th year of age	–	Cryopreservation of sperm Removal and cryopreservation of testicular tissue Storage up to 10 years
Reimbursement of GnRH analogues	No	–	No
Sufficient cost coverage of measures	No	No	Yes
Reimbursement of fertility preservation after cancer therapy	No (possibly case-by-case decision)	No	No

**Fig. 7:**
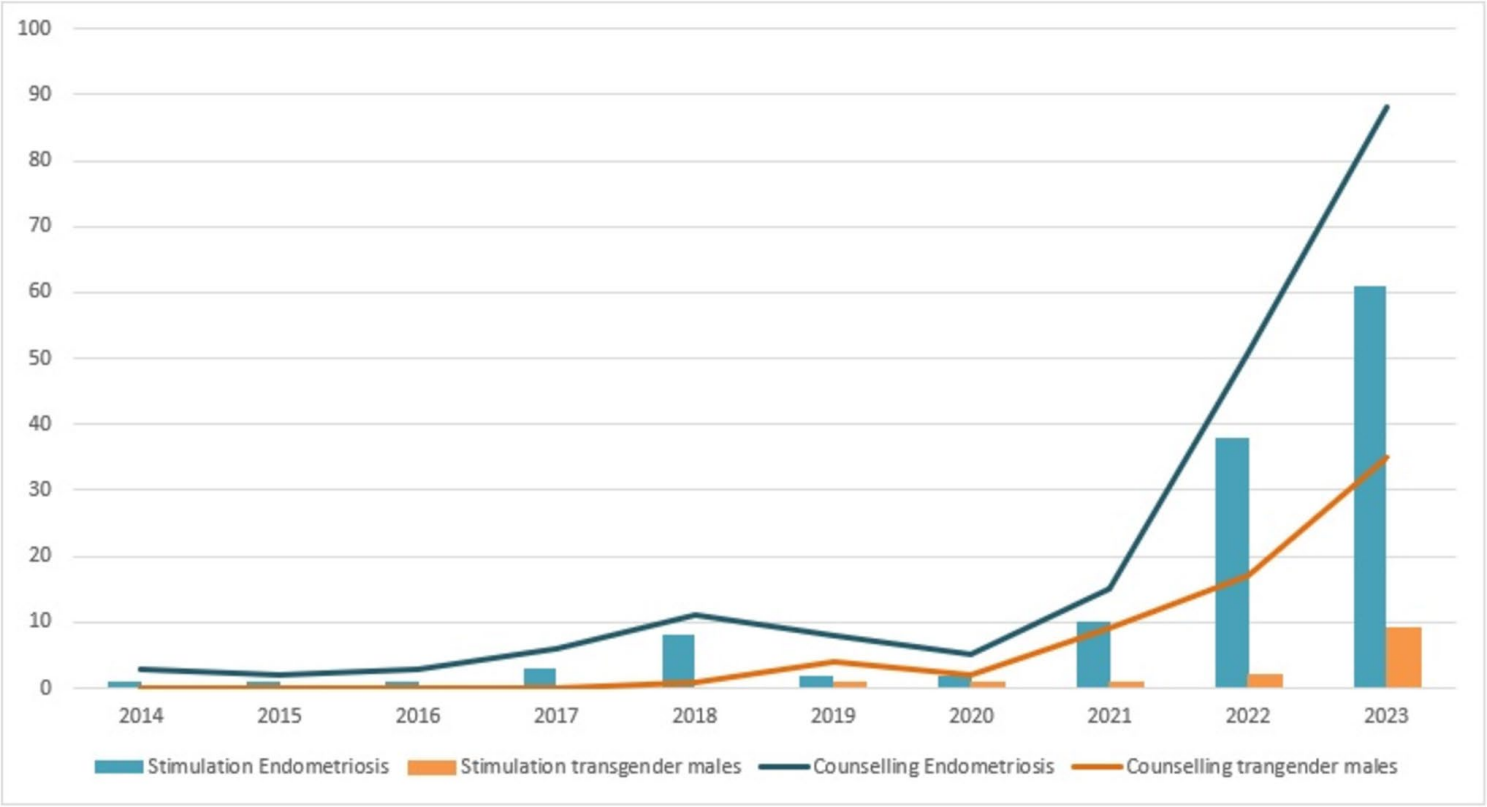
Distribution of non-malignant indications related to ovarian stimulation, data from the *Fert*iPROTEKT network (Blue bars indicate cases of endometriosis, with the blue line representing associated counselling for endometriosis. Orange represents transgender-male patients undergoing ovarian stimulation, with the orange line indicating counselling in this group; the x-axis depicts the respective years (2014–2023), the y-axis shows the total number of interventions).

Non-Oncological Indications: Several non-malignant conditions emerged as increasingly common indications for fertility preservation during the study period (Fig. 7). Endometriosis, in particular, showed a dramatic increase: Cryopreservation procedures for endometriosis rose from 4 cases (0.5% of all cryopreservation interventions) in P1 to 99 cases (5.7%) in P2—an 24-fold absolute increase and a relative increase from 0.5 to 5.7% of all procedures (*p* < 0.001). Similarly, fertility preservation procedures for transgender–male patients, which were entirely absent in P1 (0 cases), represented 11 procedures (0.6% of all cryopreservation interventions) in P2, with the majority occurring in the final 5 years of the study period (Fig. 7). These data reflect growing clinical recognition of fertility preservation as relevant to non-oncological populations and potentially increased patient awareness of preservation options.

### Comparative analysis: Period 1 (2014/2015) vs. Period 2 (2022/2023)

Key findings from the comparative analysis between 2014/2015 (P1) and 2022/2023 (P2) are summarized in Table 3.

**Table 3:** Comparison of interventions according to various criteria in two time periods 2014/2015 (P1) and 2022/2023 (P2), data from the *Fert*iPROTEKT network (data are presented as absolute numbers with corresponding percentages in brackets; y = years)

Category	2014/2015	2022/2023	P-value
All interventions	N = 1126	N = 2052	< 0.001
Public centres, n (%)	818 (72.6%)	1136 (55.4%)	
Private centres, n (%)	308 (27.4%)	916 (44.6%)	
Intervention	N = 1168	N = 2057	< 0.001
Oocyte cryopreservation, n (%)	475 (40.7%)	1427 (69.4%)	
Ovarian tissue cyropreservation, n (%)	693 (59.3%)	630 (30.6%)	
Oocyte cryopreservation	N = 475	N = 1427	0.04
< 21y, n (%)	33 (6.9%)	84 (5.9%)	
21y–30y, n (%)	222 (46.7%)	599 (42.0%)	
31y–40y, n (%)	212 (44.6%)	732 (51.3%)	
> 40y, n (%)	8 (1.7%)	12 (0.8%)	
Ovarian tissue cyropreservation	N = 693	N = 630	0.79
< 21y, n (%)	151 (21.7%)	143 (22.7%)	
21y–30y, n (%)	300 (43.3%)	266 (42.2%)	
31y–40y, (%)	233 (33.6%)	216 (34.3%)	
> 40y, (%)	9 (1.3%)	5 (0.8%)	
Oocyte cryopreservation	N = 454	N = 1427	< 0.001
Public centres, n (%)	257 (56.6%)	596 (41.8%)	
Private centres, n (%)	197 (43.4%)	831 (58.2%)	
Ovarian tissue cyropreservation	N = 672	N = 625	0.17
Public centres, n (%)	561 (83.5%)	540 (86.4%)	
Private centres, n (%)	111 (16.5%)	85 (13.6%)	
Breast cancer	N = 448	N = 943	< 0.001
Oocyte cryopreservation, n (%)	189 (42.2%)	658 (69.8%)	
Ovarian tissue cyropreservation, (%)	259 (57.8%)	285 (30.2%)	
Hodgkin lymphoma	N = 237	N = 358	< 0.001
Oocyte cryopreservation n (%)	97 (40.9%)	217 (60.6%)	
Ovarian tissue cryopreservation, (%)	140 (59.1%)	141 (39.4%)	
Non-Hodgkin lymphoma	N = 36	N = 47	0.02
Oocyte freezing, n (%)	15 (41.7%)	33 (70.2%)	
Ovarian tissue freezing, (%)	21 (58.3%)	14 (29.8%)	
Cryopreservation interventions (Oocyte and ovarian tissue cyropreservation)	N = 871	N = 1739	< 0.001
Breast cancer, n (%)	448 (51.4%)	943 (54.2%)	
Hodgkin lymphoma, n (%)	237 (27.2%)	358 (20.6%)	
Non-hodgkin lymphoma, n (%)	36 (4.1%)	47 (2.7%)	
Bone cancers	15 (1.7%)	18 (1.0%)	
Leukemia	29 (3.3%)	35 (2.0%)	
ZNS tumors	19 (2.2%)	68 (3.9%)	
Sarcoma	12 (1.4%)	21 (1.2%)	
Stomach & Colorectal diaseses cancer	29 (3.3%)	31 (1.8%)	
Other malignant disesases	13 (1.4%)	54 (3.1%)	
Endometriosis	4 (0.5%)	99 (5.7%)	
AFAB	0	11 (0.6%)	
Other non-malignant diseases	29 (3.3%)	54 (3.1%)	

Data are presented as counts and percentages; Chi-square tests were used for group comparisons. Missing centre-type data: n = 21 (4.4%) for oocyte cryopreservation (P1) and n = 21 (3.0%) for ovarian tissue cryopreservation (P1) were excluded from centre-type stratified subgroup analyses but included in overall outcome calculations. AFAB: Assigned female at birth

Shift in Procedure Type: The number of ovarian stimulation cycles increased substantially from 475 procedures in P1 to 1,427 in P2 (203% relative increase), whereas ovarian tissue cryopreservation remained relatively stable (693 vs. 630 procedures; 9% relative decrease). This shift was statistically significant (p < 0.001).

Centre Type Distribution: A pronounced shift from public to private providers was observed across all interventions (p < 0.001). For oocyte cryopreservation specifically, the proportion performed at private centres increased from 43% (n = 197/454) in P1 to 58% (n = 831/1,427) in P2 (p < 0.001). Conversely, ovarian tissue cryopreservation remained predominantly performed at public institutions in both periods (83% in P1 vs. 86% in P2; p = 0.17, not significant).

Disease-Specific Trends: Among breast cancer patients, a marked shift in procedure type was observed. Oocyte cryopreservation increased from 189 procedures (42% of breast cancer interventions) in P1 to 658 (70%) in P2 (p < 0.001), while ovarian tissue cryopreservation correspondingly decreased from 259 (58%) to 285 (30%).Similar transitions occurred in Hodgkin lymphoma (97–217 oocyte procedures, corresponding to 41–61% of lymphoma interventions; p < 0.001) and non-Hodgkin lymphoma (15 to 33 oocyte procedures, 42–70%; p < 0.05).

Age Distribution: The age distribution of oocyte cryopreservation cases showed a borderline statistically significant difference between periods (p = 0.04). While all age groups demonstrated absolute increases in procedure volume, the age structure showed minimal change: women aged 21–30 years represented 47% of oocyte procedures in P1 vs. 42% in P2, while those aged 31–40 years represented 45% in P1 vs. 51% in P2. The proportion of women > 40 years increased from 1 to 1% (8 to 12 absolute cases). For ovarian tissue cryopreservation, the age distribution remained stable (p = 0.79), with no meaningful shifts across age categories.

## Discussion

The primary aim of this study was to evaluate the evolution of medical cryopreservation strategies in the context of fertility preservation for both malignant and non-malignant diseases over the past decade, with a particular focus on ovarian stimulation and ovarian tissue cryopreservation. This was achieved by comparing two defined time periods (2014/2015 vs. 2022/2023), thereby offering a long-term view of clinical trends.

This study reveals four major findings:

First, there was a statistically significant increase in ovarian stimulation procedures, accompanied by a concurrent decrease in ovarian tissue cryopreservation. These data are consistent with a shift in clinical practice toward less invasive, more established techniques in recent years. The rise in ovarian stimulation was evident across all age groups up to 40 years, with the most pronounced increase among women aged 31–35 years, highlighting a demographic effect particularly affected by evolving preservation strategies.

Second, a decrease in ovarian tissue cryopreservation was observed across all age groups, with the exception of women under 21 years of age, in whom a moderate increase was recorded. This may reflect both a more cautious clinical approach in this cohort and the specific advantages of tissue preservation in younger individuals.

Third, ovarian tissue cryopreservation was predominantly performed in public university hospitals, while a marked increase in ovarian stimulation was observed in private fertility centres (p < 0,001). This finding may reflect differences in infrastructure, reimbursement policies, procedural complexity, and institutional experience.

Fourth, with regard to oncological indications—especially breast cancer and lymphoma—a significant increase in ovarian stimulation was observed (p < 0.001). In parallel, fertility counselling and ovarian stimulation for non-oncological (non-malignant) diseases have also increased considerably, underscoring the growing awareness and clinical application of fertility preservation in broader medical contexts.

Fertility preservation has gained substantial importance in scientific, clinical, and public discourse over the past two decades. Since the establishment of *Fert*iPROTEKT (Germany, Austria, Switzerland) in 2006, several national and international societies and networks have emerged, including the ESHRE Task and the ESHRE Special Interest group on Fertility Preservation, the Oncofertility Consortium in the USA, the International Society of Fertility Preservation (ISFP), and most recently, FertiTOX, launched in 2023. FertiTOX is dedicated to the multicenter collection and analysis of data on gonadotoxicity in both female and male patients undergoing oncological treatments. In addition, the FertiTOX consortium has published several systematic reviews addressing the gonadal toxicity associated with specific cancer therapies [4, 6, 6–10, 23].

*Fert*iPROTEKT serves as an essential tool for both clinical practice and scientific research. It provides high-quality data that reflect current trends, enables systematic follow-up of development over time [15, 26] and contributes to guideline development at the national and international level [14–16, 24]. Despite these advancements, continued monitoring and assessment of changes in clinical practices are essential, particularly as patient characteristics and medical indications evolved over time [14, 22, 24].

Since 2007, technological advancements have played a key role in shaping current fertility preservation strategies, this being mainly improvements in the efficacy, safety, and efficiency of treatments [20, 27]. The introduction and widespread adoption of vitrification as the gold standard for oocyte and embryo cryopreservation has markedly improved the outcome, replacing traditional slow-freezing techniques, due to improved survival rates [28–31]. Recently, ultra-fast vitrification and warming protocols have emerged and show encouraging results, particularly in embryo cryopreservation [31, 32]. Nevertheless, further validation through well-designed prospective studies is needed [33].

The observed increase in ovarian stimulation procedures in private clinics suggests that financial incentives in the private sector, as well as the accessibility and procedural simplicity of established workflows, may influence practice. In contrast, ovarian tissue cryopreservation remains technically and organisationally more demanding and is often centralized in the more experienced academic institutions. The stagnation in the implementation of ovarian tissue cryopreservation may reflect not merely the developmental stage of the technique but rather an interplay between its incomplete technical maturation, including challenges in cryoprotection, graft revascularization, and the lack of standardized protocols, and structural factors such as centralization of expertise, resource requirements, institutional experience, and heterogeneity in reported outcomes. Since 2019 in Switzerland [34] and since 2021 in Germany [35, 36], public health insurance reforms have allowed for partial or full reimbursement for fertility preservation (oocytes, sperm, or gonadal tissue) in patients at risk of infertility due to oncological treatments such as chemotherapy, radiotherapy, or surgical interventions. The temporal coincidence of these policy reforms with increased intervention numbers suggests a potential association; however, multiple factors may have influenced practice patterns [37].

Our findings confirm a consistent and gradual increase in ovarian stimulation under the age of 40, especially in the 31–35 age group. The observed shift is likely driven by greater insurance coverage, improved success rates, and increased patient awareness.

In paediatric, adolescents and young adult patients, ovarian tissue cryopreservation remains the method of choice. This is attributed to the high ovarian reserve at a young age and the inapplicability of stimulation protocols in prepubertal individuals [13]. Furthermore, differences in fertility preservation technique selection appear to be influenced by disease-specific considerations. For example, in patients with lymphoma, which is the second most prevalent form of cancer in these young patients, both tissue and oocyte preservation are common, but favouring ovarian tissue preservation due to the clinical and therapeutic characteristics of the disease. Lymphoma often requires the immediate initiation of chemotherapy, leaving limited time for controlled ovarian stimulation and oocyte retrieval. In contrast, ovarian tissue cryopreservation can be performed without delaying oncological treatment and allows the preservation of numerous primordial follicles in a single procedure [38]. Several studies have demonstrated its safety and efficacy in this setting, although caution is advised due to the potential risk of malignant cell reintroduction, particularly in hematological malignancies [39]. Therefore, histological and molecular screening of ovarian tissue is recommended prior to transplantation [40]. Variability in reported success rates likely reflects differences in institutional experience, processing protocols, and patient selection, underscoring the need for standardized methodologies and the concentration of expertise in specialized centers.

Our data also show a growing number of non-oncological indications for fertility preservation [3, 41]. This includes endometriosis, a chronic gynaecological condition affecting approximately 10% of women of reproductive age, and is now a leading indication for elective cryopreservation [41–46]. In cases with severe ovarian involvement or repeated surgeries, the resulting decrease in ovarian reserve justifies early vitrification of oocytes [47–49]. Furthermore, women with genetic mutations in the BRCA1- and BRCA2-associated hereditary breast and ovarian cancer (HBOC) syndrome at risk of developing breast and ovarian cancer [50–52] opt for oocyte cryopreservation prior to a prophylactic oophorectomy at an early age. The increased consultations and interventions underscore the role of fertility preservation as a preventive strategy attributed to significant advances in the detection of early stages and the development of genetic testing indications.

The rise in gender incongruence diagnosis, particularly among adolescents and young adults, has further expanded the scope of fertility preservation. Current recommendations advise that fertility preservation options should be discussed with transgender-male patients prior to Gender Affirming Medical Treatment with the initiation of hormone therapy or irreversible surgical interventions. The option of cryopreserving their gametes or gonadal tissue should take into account both biological viability and ethical and psychosocial aspects [53, 54]. Our findings reflect this trend, with a noticeable increase in both consultations and ovarian stimulations for transgender-male patients, especially in the past 5 years.

During the initial phase of the COVID-19 pandemic, restrictive hospital measures led to a temporary suspension of elective procedures like fertility preservation [18, 19, 55]. The suspension had a significant impact on patients with non-malignant indications and those who opted to undergo elective oocytes cryopreservation [56]. Our data show a substantial decline in cryopreservation procedures for non-oncological elective indications between 2019 and 2020. Nonetheless, urgent oncological cases were largely managed through adapted clinical protocols and pathways, thereby reinforcing the recognition of fertility preservation as a medical necessity. The prevailing priority was allocated to ovarian stimulation for malignant diseases rather than the cryopreservation of ovarian tissue. Following the pandemic, there has been a resurge in demand, driven by heightened public awareness of health-related uncertainty in reproductive planning and resulting in an increase in medical and non-medical consultations.

The strength of this study lies in the large dataset encompassing both ovarian stimulation and tissue cryopreservation cases over a ten-year period. The inclusion of data from an international, tri-national registry (*Fert*iPROTEKT) ensures a high level of representativeness, covering a wide range of centers, from university hospitals to private clinics. This heterogeneity supports a more comprehensive understanding of real-world clinical practices. Another strength is the study’s multicenter design, which enhances the generalisability of the findings and allows for the detection of trends that are not limited to individual institutions or health care systems. Moreover, the comparison between two time periods provides valuable insight into changes over time.

However, some limitations must be acknowledged. In our registry data, certain relevant variables were inaccessible or incomplete, such as detailed chemotherapy regimens, hormonal status at baseline, and long-term fertility outcomes, which may have affected the robustness of the subgroup analysis. Further limitations include the variation in practice across participating centers and countries, including differences in legal frameworks, counselling standards, and reimbursement policies.

Further research is needed to explore the expanding landscape of non-oncological fertility preservation, including patients with endometriosis, autoimmune diseases, genetic syndromes, and transgender-male persons. Although these indications are becoming more prevalent and represent an increasing proportion of fertility preservation interventions, long-term data remain limited. Continuous and systematic follow-up through structured registries such as FertiTOX will be critical for determining how many individuals experience gonadotoxic effects and return to use their cryopreserved material [23]. Consequently, long-term success and safety of various cryopreservation strategies has to be assessed. In the context of emerging cancer therapies, systematic assessment of gonadotoxic effects will be essential for risk stratification, clinical counselling, and development of personalized protocols. Robust outcome data will not only inform clinical decision-making but also guide public health planning and ensure equitable access to fertility preservation.

## Conclusion

Fertility preservation plays an increasingly pivotal role in reproductive medicine. Our study demonstrates a clear shift in clinical practice over the current decade with a rise in ovarian stimulation and a decline in ovarian tissue cryopreservation, alongside an expansion in indications and a broader spectrum of patients. These developments are influenced by technological progress, policy reforms, and a growing awareness among patients and clinicians. The establishment and continued optimisation of national and international fertility preservation programmes or networks, combined with supportive reimbursement structures, are essential to meeting future needs. A deep understanding of evolving trends and the ability to adapt will be crucial to ensure that fertility preservation remains a fundamental and equitable element of modern reproductive medicine. These trends include fertility preservation strategies in response to advances in oncological treatments, changing patient demographics, and growing ethical and policy considerations requiring continued multidisciplinary adaptation.

## Supplementary Information

Below is the link to the electronic supplementary material.

Supplementary file 1 (DOCX 112 KB)

## Data Availability

The datasets generated and/or analyzed during the current study are available from the corresponding author upon reasonable request.
